# The Cause of Unexpected Acute Abdomen and Intra-Abdominal Hemorrhage in 24-Week Pregnant Woman: Bochdalek Hernia

**DOI:** 10.1155/2016/6591714

**Published:** 2016-11-27

**Authors:** Yavuz Savas Koca, Ibrahim Barut, İhsan Yildiz, Rasih Yazkan

**Affiliations:** ^1^Department of General Surgery, School of Medicine, Süleyman Demirel University, Isparta, Turkey; ^2^Department of General Surgery, School of Medicine, HPB Surgery Unit, Süleyman Demirel University, Isparta, Turkey; ^3^Department of Thorax Surgery, School of Medicine, Süleyman Demirel University, Isparta, Turkey

## Abstract

Bochdalek hernia (BH) is the most common type of congenital diaphragm hernia and is rarely seen in adults. In adult patients, BH often remains asymptomatic or presents with nondiagnostic symptoms and may lead to complications, though rarely. The necrosis and perforations occurring in the hernia may lead to mortality. In this report, we present a 34-year-old pregnant woman at 24 gestational weeks who presented with Bochdalek hernia causing gastric volvulus associated with perforation and intra-abdominal hemorrhage associated with splenic rupture.

## 1. Introduction

Bochdalek hernia is typically known as a neonatal disease and is rarely seen in pregnant adults. In adult patients, it often remains asymptomatic. The reported incidence of incidental BH is 0.17% [1] and the incidence of BH in pregnant adults is even rarer. Bochdalek hernia is difficult to diagnose due to nonspecific symptoms and the diagnosis of BH is sometimes established by the clinical manifestation of complications. The presence of complications indicates high rates of mortality and morbidity. Commonly reported complications include gastric, colonic, and esophageal necrosis and omentum hemorrhage leading to hemothorax. However, no splenic rupture caused by recessed location of the spleen has been reported in the literature. In this report, we present a 34-year-old pregnant woman with Bochdalek hernia causing total gastric necrosis, gastrointestinal perforation, and splenic rupture.

## 2. Case Report

A 34-year-old female patient at 24 gestational weeks presented to our emergency service with the complaints of vomiting, abdominal pain, weakness, and dyspnea. The patient had been suffering from vomiting for the last 10 days and from dyspnea and weakness for the last 3 days. The patient had previously presented to the hospital several times with the complaints of dyspnea and abdominal pain but had no diagnosis. The patient had a live birth delivery via caesarian section after 2 uneventful pregnancies. Physical examination revealed reduced breath sounds on the left side of the chest and diffuse abdominal tenderness and rebound tenderness on palpation. Arterial blood pressure was 70/50 mmHg, pulse rate was 118/min, body temperature was 37.8°C, and respiratory rate was 25/min. During ultrasonographic examination of the patient, the patient had cardiopulmonary arrest. The patient was resuscitated by cardiopulmonary resuscitation in approximately 7 minutes and then was intubated and transferred to the intensive care unit.

**Figure 1:**
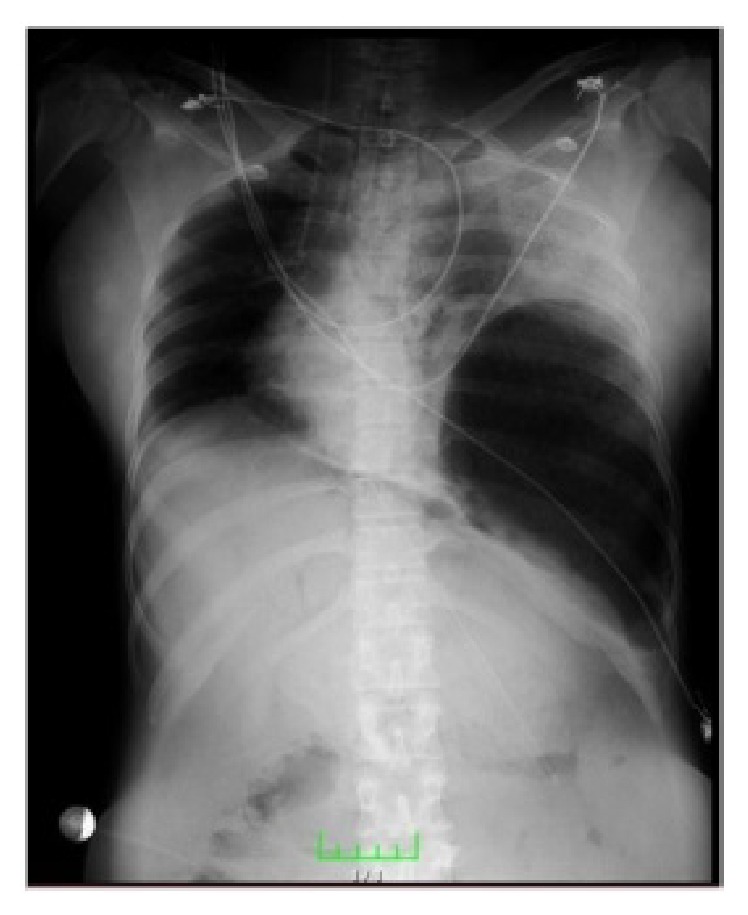
X-ray subdiaphragmatic free gas.

Laboratory parameters revealed white cell count of 21,000/mL, hemoglobin of 7.4 g/dL, platelet count of 31,8000/mL, AST of 64 U/L, ALT of 74 U/L, creatinine of 1.5 mg/dL, and C-reactive protein of 118 mg/L. A chest X-ray was performed in the intensive care unit and showed an impression suggestive of free air under the left hemidiaphragm; therefore, a left lateral decubitus abdominal radiograph was performed and the anterior posterior image showed that the free air moved to the right side (Figure 1). During the surgical decision-making process, an emergency obstetric USG examination was performed and it revealed intrauterine fetal demise.

**Figure 2:**
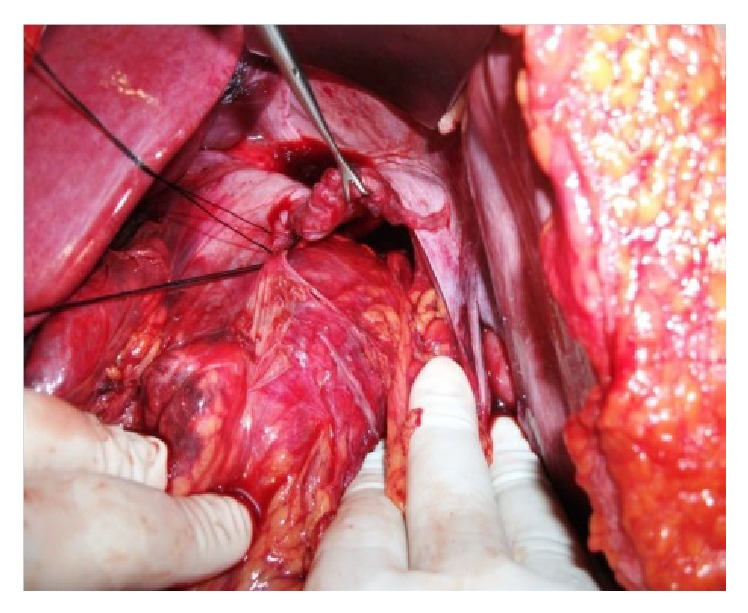
Posterior diaphragmatic defect (14 × 8 cm).

The patient was operated on under emergency conditions. During laparotomy, there was gush of air during the initial excision of the peritoneum and 2,400 cc of blood was transfused during intra-abdominal exploration. A 14 × 8 cm defect was detected in the posterior diaphragm (Figure 2).

**Figure 3:**
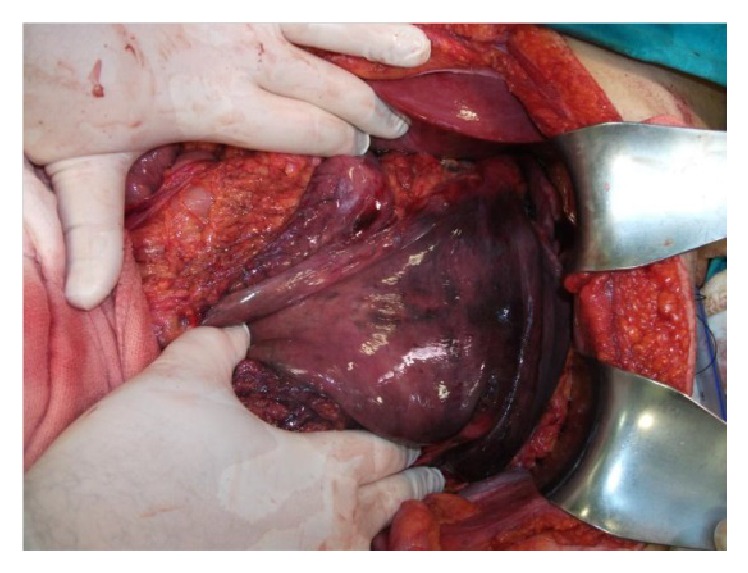
Total necrotic stomach.

The spleen was at the medial aspect of the abdomen and the hilum was actively bleeding. No splenectomy was performed. The stomach and the omentum in the left hemothorax were reduced back manually into the abdomen. The stomach was completely necrotic and the lesser curvature perforation was detected on the anterior aspect of the corpus (Figure 3).

A large hernia sac was detected in the diaphragmatic cavity. The defect in the posterior diaphragm was closed by primary repair following the insertion of a gastrostomy tube. After the improvement of hemodynamics and oxygen saturation of the patient, the dead fetus was delivered via caesarian section. Subsequently, total gastrectomy followed by Roux-en-Y anastomosis was performed. Postoperative follow-up of the patient was performed in the intensive care unit. On the 27th day of the treatment, the patient died due to brain hypoxia caused by cardiopulmonary arrest.

## 3. Discussion

Bochdalek hernia is the most common type of congenital diaphragm hernia (CDH) (90%), followed by Morgagni hernia (1–5%) and esophageal hernia (1–5%) [2]. BH is mostly localized in the left posterolateral aspect of the diaphragm (80–90%) and is twice more common in men than in women. Similarly, in our patient, the defect was located in the left side of the diaphragm. Bochdalek hernia often results from the herniation of abdominal organs into the thoracic cavity as a result of insufficient closure of the fusion between the posterolateral pleuroperitoneal membrane and the septum transversum [3]. Although BH is the most common type of CDH, it is rarely seen in adults. However, some clinical conditions that elevate intra-abdominal pressure in adults, such as pregnancy, further increase the complication rate in BH [4].

Bochdalek hernia often presents with nonspecific symptoms. These symptoms may vary greatly, depending on the organs involved and the presence of strangulation. Although dyspnea and tachypnea are the most common presenting symptoms, patients with gastric volvulus often present with abdominal pain and vomiting. The case presented in this study had previously presented to the hospital several times with the complaints of dyspnea and abdominal pain but had no diagnosis. The literature shows that mortality has been reported in some of the patients with late diagnosis of BH [5]. Our patient presented to our emergency service with abdominal pain, nausea, vomiting, and dyspnea and had cardiopulmonary arrest during the initial examination. The patient was resuscitated by cardiopulmonary resuscitation but died on the 27th day of the treatment.

X-ray is commonly used for establishing the diagnosis of BH but computed tomography is the gold standard in the diagnosis of BH. Computed tomography is highly useful in the visualization of the organs in the hernia sac in the diaphragmatic cavity as well as the clinical condition of the organs and the lungs. Our patient was immediately operated on under emergency conditions following the resuscitation due to the detection of free air in the left hemidiaphragm. In the literature, severe hemorrhage caused by the protrusion of the omentum in the thoracic cavity has been reported in patients with CDH [6]. However, similar to our patient, no hemorrhage caused by splenic laceration has been reported [7].

Laparoscopic repair and mesh repair, particularly in large defects, are the methods of choice in the treatment of BH [8]. In our patient, laparotomy was performed since the patient was unstable.

Bochdalek hernia is less common in adults compared to infants, and once the diagnosis has been established, BH should be operated on under elective conditions since it may lead to fatal complications in later stages.
